# Food availability, temperature, and day length drive seasonal variations in the positional behavior of white‐headed langurs in the limestone forests of Southwest Guangxi, China

**DOI:** 10.1002/ece3.8171

**Published:** 2021-10-05

**Authors:** Jingjin Zheng, Kechu Zhang, Jipeng Liang, Youbang Li, Zhonghao Huang

**Affiliations:** ^1^ Key Laboratory of Ecology of Rare and Endangered Species and Environmental Protection (Guangxi Normal University), Ministry of Education Guilin China; ^2^ Guangxi Key Laboratory of Rare and Endangered Animal Ecology Guangxi Normal University Guilin China; ^3^ Administration Center of Guangxi Chongzuo White‐Headed Langur National Nature Reserve Chongzuo China

**Keywords:** food availability, positional behavior, thermoregulation, white‐headed langur

## Abstract

Information on positional behavior contributes to the understanding of the ecological adaptation mechanisms in animals. We collected data on the positional behavior of white‐headed langurs (*Trachypithecus leucocephalus*) at the Guangxi Chongzuo White‐Headed Langur National Nature Reserve from September 2016 to August 2017 *via* instantaneous scan sampling method. This study aimed to examine the importance of positional behavior flexibility in limestone forests characterized by seasonal variations in climate and food resources. Our results indicated that langurs adopted leaping (47.92% ± 5.50%) and vertical climbing (40.13% ± 6.20%) as their predominant locomotor modes and sitting (83.08% ± 4.70%) as their predominant posture. Their positional behavior exhibited marked seasonal variations. More specifically, langurs used quadrupedal walking more frequently during the dry season than during the rainy months. In the stationary state, they sat more frequently during the dry season, whereas they laid and suspended more often during the rainy season. Their positional behavior was affected by fruit availability, day length, and temperature. Quadrupedal walking increased with the decrease in fruit availability, whereas leaping was positively correlated with fruit availability. Moreover, sitting was positively correlated with average temperature but negatively correlated with day length. Lying was also negatively correlated with temperature but positively correlated with day length. We conclude that white‐headed langurs adapt to limestone forests with positional behavior flexibility in response to seasonality. Our research provides evidence of the effects of food availability, ambient temperature, and day length on the positional behavior of white‐headed langurs, highlighting the need to understand their behavioral ecology and the influence of ecological factors on behavioral adaptation.

## Graphical abstract

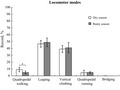

## 1 INTRODUCTION

Positional behavior contributes to the understanding of animal ecological adaptation mechanisms (Fleagle, 2013). It includes locomotor modes and postures (Hunt et al., 1996; Prost, 1965), which are related to how animals move through their environments, acquire food, avoid predators, and socially communicate (Cant, 1992; Gebo et al., 1994; Larson, 2018). Positional behavior is linked to morphological and physiological characteristics, such as body size (Gebo, 1992; Hunt, 1994; Mcgraw, 1998), limb length (Chen et al., 2020; Fleagle, 2013; Fleagle & Meldrum, 1988; Huang, Huang, Wei, et al., 2015; Wright, 2007), and tail length (Chatani, 2003; Fleagle, 2013; Huang, Huang, Wei, et al., 2015). Moreover, it is influenced by ecological factors, including habitat structure (Dagosto & Yamashita, 1998; Garber & Pruetz, 1995; Huang, Huang, Wei, et al., 2015), the spatial distribution of food resources and seasonal variation (Chen et al., 2020; Dagosto, 1995), and climatic factors (Dasilva, 1993; Mandl et al., 2018).

Morphological and anatomical characteristics play significant roles in shaping the positional behavior of primates (Fleagle, 2013; Gebo, 1992; Huang, Huang, Wei, et al., 2015; Mcgraw, 1998; Wright, 2007). Generally, the frequency of animals’ locomotor modes is related to body size (Fleagle, 2013). For instance, the smaller species among the seven sympatric Surinam monkeys are more likely to cross discontinuities by leaping, whereas larger species are more likely to cross gaps by climbing or bridging (Fleagle & Meldrum, 1988). This may be because leaping requires great propulsive forces generated from the hind limbs, such that larger animals require a greater force than smaller animals (Fleagle, 2013). A similar pattern has been observed in the locomotor modes of sympatric macaques (Huang, Huang, Wei, et al., 2015). The Intermembral Index (IMI, ratio of forelimb length to hind limb length) is also correlated with the locomotor modes of primates (Fleagle, 2013). More specifically, primates with a lower IMI leap most frequently when traveling, as reported in François’ langurs (*Trachypithecus. francoisi*; Chen et al., 2020) and Indo‐Chinese gray langurs (*T. crepusculus*; Ma & Fan, 2020). Contrarily, compared with primates with lower IMIs, primates with higher IMIs, such as Cao Vit gibbons (*Nomascus nasutus*; Fan et al., 2013), red‐shanked doucs (*Pygathrix nemaeus*), and gray‐shanked doucs (*P. cinerea*; Wright et al., 2008), are skilled at suspensory locomotion. Moreover, other primates with intermediate IMIs tend to most frequently use walking and climbing when moving, as observed in Assamese macaques (*Macaca assamensis*) and rhesus macaques (*M. mulatta*; Huang, Huang, Wei, et al., 2015). In addition, tail length is also related to leaping ability (Fleagle, 2013): A long tail, which may function as a balancer, helps animals leap across longer distances (Chatani, 2003).

Ecological factors, such as habitat structure, food availability, and climatic factors, also play critical roles in shaping the positional behavior of primates (Dagosto, 1995; Fleagle, 2013; Garber & Pruetz, 1995; Kelley et al., 2016). Among many ecological factors, food‐related factors are thought to have the greatest impact on positional behavior because seasonal variations in food resources result in seasonal variations in positional behavior in wild environments (Dagosto, 1995; Youlatos, 1998). More specifically, for the purpose of reducing the expenditure of energy, maximizing foraging benefits, and reducing predation risk, primates adopt different positional behaviors to harvest foods (Cant, 1992; Gebo et al., 1994). For instance, when leaves become the dominant food resource during the dry season, red howling monkeys (*Alouatta seniculus*) more frequently use quadrupedal walking while traveling, likely because walking quadrupedally on large substrates is energetically inexpensive and relatively stable (Youlatos, 1998). During the wet season when fruits are abundant, red howling monkeys more frequently sit during feeding (Youlatos, 1998), likely because fruits are abundant, enabling primates to consume food while sitting. Habitat structure is another important factor influencing primates’ positional behavior (Gebo & Chapman, 1995a; Huang, Huang, Wei, et al., 2015). For instance, the frequency of various locomotor modes in red colobus monkeys (*Colobus badius*) varies across forests (Gebo & Chapman, 1995a). Differences in the locomotor pattern in langurs across different limestone forest primates may be linked to variations in habitat (Chen et al., 2020; Workman & Schmitt, 2012; Zhou et al., 2013).

Temperature also influences the positional behavior of primates (Kosheleff & Anderson, 2009). To ensure optimal body functioning, mammals maintain a constant body temperature via behavioral thermoregulation (McFarland et al., 2020). When temperatures fluctuate, the physiological mechanisms that keep core temperature within the thermal neutral zone are enhanced, resulting in increases in the energy expenditure rates (Terrien et al., 2011). Luckily, behavioral adjustments can reduce the energy expenditure caused by the thermoregulatory responses of animals (Li et al., 2020; Terrien et al., 2011). To adapt to environmental temperature changes, primates usually adjust their positional behavior to minimize metabolic costs (Aujard et al., 2006). For instance, primates use huddling, curling, and basking in the sun when ambient temperatures are low (Donati et al., 2011; Fan et al., 2012; Kelley et al., 2016; Li, Huang, et al., 2020; Zhou et al., 2007) and shading or exposing their back for heat dissipation when ambient temperatures are high (Bicca‐Marques & Calegaro‐Marques, 1998; Campos & Fedigan, 2009; McFarland et al., 2020).

White‐headed langurs (*Trachypithecus leucocephalus*) are exclusively distributed in the limestone forests of Southwest Guangxi, China (Huang, 2002). These langurs are typically folivorous, preferring to eat young leaves (Huang et al., 2017; Zhang et al., 2017). Limestone forests are characterized by vertical cliffs and seasonal limestone rainforests, where most trees are vertically distributed, but canopies are largely not contiguous (Fan et al., 2011). Langurs select ledges and caves in the middle and upper cliffs as sleeping sites (Huang, 2002). Moreover, limestone forests are characterized by high plant species diversity with low biomass (Huang et al., 2010) and demonstrate marked seasonal variations in food availability, ambient temperature, and rainfall (Huang, 2002; Huang, Huang, Tang, et al., 2015; Li et al., 2020b). During the dry season, when food availability and temperatures are low, these langurs spend more time feeding (Zhou et al., 2010) and tend to sunbathe on bare rocks (Huang, 2002). During the rainy season, when temperatures are high, these langurs prefer to rest in the forest (Huang, 2002). Studies demonstrate that the behavior of white‐headed langurs may be related to food availability and climate (Li & Rogers, 2006; Zhang et al., 2021; Zhou et al., 2011), suggesting that their positional behavior may be influenced by these ecological factors. In this study, we first describe the positional behavior of white‐headed langurs, and then focus on seasonal variations in this behavior. Finally, we discuss the effects of ecological factors on the positional behavior of white‐headed langurs by testing the following predictions:

1. White‐headed langurs have relatively low IMIs (76) (Huang & Li, 2005; Pan et al., 1989) and inhabit limestone forests characterized by limestone seasonal rainforests and vertical cliffs (Huang, 2002). Therefore, we predict that leaping and vertical climbing are the predominant locomotor modes for these langurs.
2. The food choices of white‐headed langurs are influenced by food availability (Lu et al., 2016). These langurs prefer to feed on young leaves and consume leaves almost year‐round (Li & Rogers, 2006). To harvest enough young leaves, they spend more time searching for food during the dry season (Zhou et al., 2010). Quadrupedal walking is considered to be an energy‐saving positional behavior (Nakatsukasa et al., 2006). As such, to reduce energy expenditure and maximize foraging benefits, we predict that these langurs use quadrupedal walking more frequently during the dry season than during the rainy season.
3. Previous studies indicate that white‐headed langurs like to spend time in the shade during the rainy season and bask in the sun during the dry season (Huang, 2002; Huang & Lu, 1993). Sunning, huddling, and curling are effective strategies for minimizing the loss of body heat, whereas extended postures can effectively dissipate body heat during the hot season (Donati et al., 2011). Mean temperatures in the rainy season are higher than those in the dry season (bare rock: 39.2°C versus 29.1°C; forest: 25.0°C versus 20.0°C; Zhang et al., 2021). Thus, we predict that langurs sit together (huddle) more frequently during the dry season and lay more frequently during the rainy season.

## 2 METHODS

### 2.1 Study site and subjects

This research was conducted in the Guangxi Chongzuo White‐Headed Langur National Nature Reserve (107°16′53″–107°59′46″E, 22°10′43″–22°36′55″N), Southwest Guangxi, China. The reserve consists of four parts: Banli, Tuozhu, Bapen, and Dalin. This research site is located in the Banli district, with a total area of 28.3 km^2^. The reserve is covered by karst limestone hills with an elevation ranging from 400 to 600 m and is characterized by a limestone seasonal rainforest (Guangxi Forestry Department, 1993). Due to human activities, the habitat is severely fragmented (Huang et al., 2008). During the research period, the total rainfall was 4,382.9 mm, and the mean annual temperature was 24.3°C (Zhang et al., 2020). Based on the monthly rainfall, the research period was divided into a dry season from September to February and a rainy season from March to August (Zhang et al., 2020).

During the study period, four groups of white‐headed langurs were found. At the beginning of the study, 42 individuals from the four groups were represented as follows: Dushan group (G‐DS; *n* = 15, 1 adult male, 13 adult females, and 1 subadult), Zhiwuyuan group (G‐ZWY; *n* = 16, 1 adult male, 9 adult females, and 6 infants), Leizhai group (G‐LZ; *n* = 6, 1 adult male and 5 adult females), and Nanong group (G‐NN; *n* = 5, 1 adult male and 4 adult females). Detailed information on group sizes during the study is described by Zhang et al. (2020).

### 2.2 Behavioral data collection

From September 2016 to August 2017, we used spotting scopes and binoculars to observe the white‐headed langurs and collected behavioral data using instantaneous scan sampling method, with a 5‐min scanning period and a 10‐min subsequent interval (Altmann, 1974). We scanned these langurs from left to right (or in a clockwise direction) to avoid scanning bias toward certain individuals. During a scan, we collected behavioral records of as many different individuals as possible, but each individual was only sampled once (Huang, Huang, Wei, et al., 2015). After 5 s of observation, we recorded the predominant behavior of each scanned individual, including resting, moving, feeding, and social grooming. Detailed descriptions of the behavioral data collection methodology are described by Zhang et al. (2020).

During scanning, the positional behavior of each subject individual was recorded. Positional behavior was divided into five locomotor modes (quadrupedal walking, leaping, vertical climbing, quadrupedal running, and bridging) and four postures (sitting, lying, quadrupedal standing, and suspending). The definitions of locomotor and postural modes were based on Hunt et al. (1996; Table 1). During scanning, we also recorded the substrate type used by the langurs, including trees, shrubs, lianas, bare rocks, and flat land.

**TABLE 1:** Definitions of positional behavior in this study, following Hunt et al. (1996)

Terms	Definitions
Locomotor modes	
Quadrupedal walking	All four limbs contacting the substrate angled less than 45°, and the trunk is pronograde or roughly parallel
Leaping	A gap‐crossing movement in which the hind limbs principally function as propulsors
Vertical climbing	Moving up or down a vertical or steeply inclined substrate
Quadrupedal running	Rapid movement with an asymmetrical or irregular gait, with a period of free‐flying
Bridging	Movement of crossing gaps where the hands reach out to grasp a support on one side of a gap and cautiously pulling the body across the open space with the feet, never involving an airborne phase
Postures	
Sitting	The trunk is perpendicular to the substrate, and the ischia and ischial callosities primarily bear the body weight
Lying	Posture that the ventral, dorsal, or side aspect of trunk supports body weight
Quadrupedal standing	All limbs standing on horizontal or subhorizontal substrate; the elbow and knee are straight and the trunk is nearly horizontal
Suspending	Stationary posture that limbs or tail hangs on the support, such as hind limb suspension

### 2.3 Food availability assessment

According to a previously described method for food availability assessment (Huang, Huang, Tang, et al., 2015), we randomly selected 270 plants of 27 species (10 individuals of each species) for phenological monitoring. In the middle of each month, we visually inspected the tagged trees entirely for the presence of young leaves, mature leaves, flowers, and fruits and scored the trees according to their relative abundance on a six‐point scale (0–5 points). The monthly food availability index for specific items was calculated by integrating the canopy volume and phenology score of the sampled trees. The calculation formula is as follows:

$$\text{FAI}=\sum_{i=1}^{n}V_{i}P_{i},$$

where *V_i_* denotes the canopy volume of species *i*, and *P_i_* denotes the assignment values of different food parts of species *i* (Zhang et al., 2021).

### 2.4 Data analysis

Behavioral records of dependent infants were excluded from the analysis because they neither moved nor traveled independently. We conducted statistical analysis for each group separately, with results expressed at the group level because individual identifications were not possible due to the observation distances. More specifically, we obtained the total scans for each month by merging all data from all scanned individuals. Based on the monthly total samples for locomotor and postural modes, we obtained monthly percentages for each behavior. Then, we obtained annual and seasonal percentages by averaging the relevant monthly values. In addition, we assessed substrate utilization using a similar method. Based on the monthly total samples for specific behaviors, we obtained the monthly percentages of each behavior across the substrate types. Then, we compared the monthly percentages across substrate types using the Kruskal–Wallis test with a *post hoc* test of pairwise comparisons.

To improve linearity, numeric variables such as food availability (fruit availability, flower availability, young leaf availability, and mature leaf availability), temperatures (rock maximum temperature and forest average temperature), rainfall, and day length were log_10_(X)‐transformed (Li et al., 2020), whereas the variables expressed in percentages, including locomotor modes, postural modes, and humidity (rock relative humidity and humidity under the forest), were logit‐transformed (Li, Ma, Zhou, Li, et al., 2020; Warton & Hui, 2011).

In accordance with a previous study (Huang et al., 2017), we developed generalized linear mixed models (GLMMs) to examine the differences in the positional behaviors of white‐headed langurs between the rainy and dry seasons. We set the positional behaviors as the response variables, season as a fixed factor, and sample sizes and groups (G‐DS, G‐ZWY, G‐LZ, and G‐NN) as random factors. Furthermore, we conducted an ANOVA to test the effects of season on each special variable by comparing the models with and without fixed factors. Season significantly shaped the goodness‐of‐fit of the models when the *p* value was <.05, suggesting that the response variables had significant variation across seasons (Huang et al., 2017; Zhang et al., 2020).

Following Huang et al. (2017) and Li, Ma, Zhou, Li, et al. (2020), we constructed GLMMs to examine the influence of ecological factors on positional behavior. The percentage of specific positional behaviors was considered as the response variable, whereas food availability (including the availability of young leaves, flowers, fruits, and mature leaves) and climatic factors (including temperature, day length, rainfall, and humidity) were set as explanatory variables, and group (G‐DS, G‐ZWY, G‐LZ, and G‐NN) was considered to be a random effect. We used the average temperature under the forest and the maximum rock temperature as a proxy for temperature, since the correlation coefficients between these temperatures and other ecological factors were small. We considered models in which the difference in the AICc values between each model and the best‐ranked model (lowest AIC) was less than 2 (∆AIC ≤ 2) as the highly supported models (Li, Ma, Zhou, Li, et al., 2020). The relative importance of each predictor (W*_ip_*) included in the highly supported model was obtained by summing the Akaike weights (W*_i_*) for each model (Burnham & Anderson, 2002; Li, Ma, Zhou, Li, et al., 2020). The predictors contained in highly supported models were the most important factors affecting the response variables when their 95% confidence intervals for *β*‐values excluded zero (Burnham & Anderson, 2002; Li, Ma, Zhou, Li, et al., 2020). We conducted GLMMs using the *lmer* function in the *lme4* package (Bates et al., 2015) and the *model.avg* function in the *MuMIn* package (Bartoń, 2019) in R 4.0.4 (R Core Team, 2021). All tests were two‐tailed, and significance levels were set to 0.05.

## 3 RESULTS

### 3.1 General locomotor patterns and seasonal differences

**FIGURE 1:**
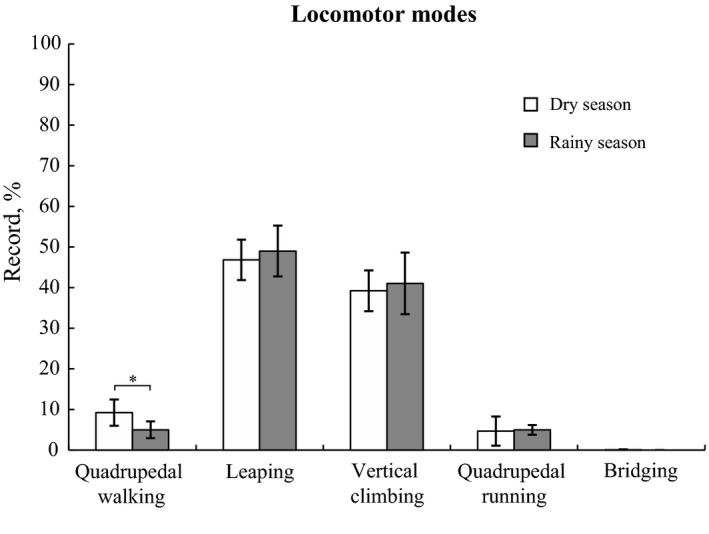
Frequencies of locomotor modes used by the white‐headed langurs at Guangxi Chongzuo White‐Headed Langur National Nature Reserve from September 2016 to August 2017. Asterisk represents a significant difference between the dry and rainy seasons: *p < .05

Leaping was the most common locomotor mode for white‐headed langurs (47.92% ± 5.50% of annual records), followed by vertical climbing (40.13% ± 6.20%), quadrupedal walking (7.10% ± 3.41%), quadrupedal running (4.82% ± 2.56%), and bridging (0.03% ± 0.07%; Table 2). Langurs performed quadrupedal walking more frequently during the dry season (*χ* ^2^ = 12.747, *df* = 1, *p* < .001) than during the rainy season; however, there was no significant seasonal difference in other modes of locomotion (leaping: *χ* ^2^ = 0.608, *df* = 1, *p* = .436; vertical climbing: *χ* ^2^ = 0.580, *df* = 1, *p* = .447; quadrupedal running: *χ* ^2^ = 1.985, *df* = 1, *p* = .159; bridging: *χ* ^2^ = 2.251, *df* = 1, *p* = .134; Figure 1).

**TABLE 2:** Frequencies of locomotor modes and postures used by the white‐headed langurs (% of record)

Locomotor modes	Quadrupedal walking	Leaping	Vertical climbing	Quadrupedal running	Bridging
	7.10 ± 3.41	47.92 ± 5.50	40.13 ± 6.20	4.82 ± 2.56	0.03 ± 0.07

Postures	Sitting	Lying	Quadrupedal standing	Suspending	
	83.08 ± 4.70	16.16 ± 4.56	0.21 ± 0.17	0.55 ± 0.31	

### 3.2 General postural patterns and seasonal variations

**FIGURE 2:**
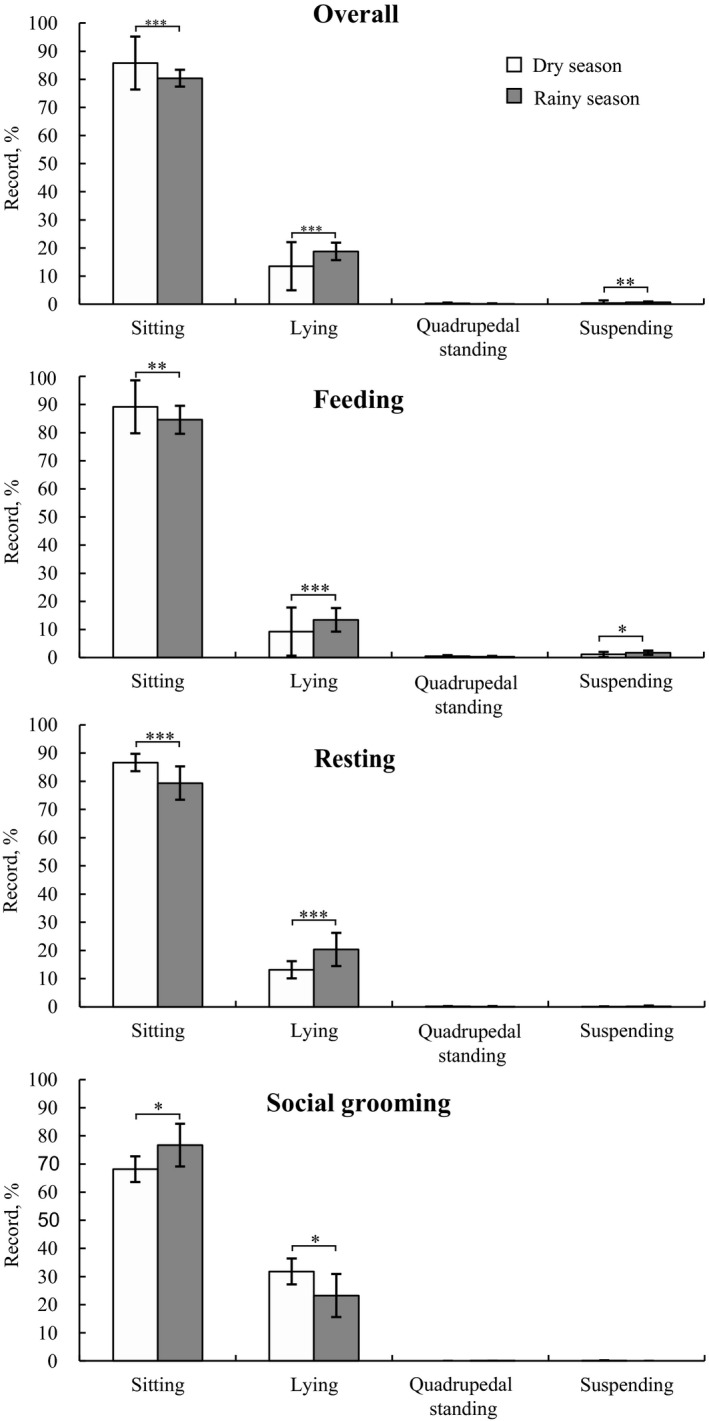
Frequencies of postures during overall activity, resting, feeding, and social grooming used by the white‐headed langurs at Guangxi Chongzuo White‐Headed Langur National Nature Reserve from September 2016 to August 2017. Asterisks represent significant differences in proportions between the dry and rainy season: ***p < .001; **p < .01; *p < .05

Overall, sitting was the most frequently used posture for white‐headed langurs (83.08% ± 4.70% of annual records), followed by lying (16.16% ± 4.56%), quadrupedal standing (0.21% ± 0.17%), and suspending (0.55% ± 0.31%; Table 2). Langurs sat more frequently during the dry season (*χ* ^2^ = 14.018, *df* = 1, *p* < .001), whereas they lay (*χ* ^2^ = 14.421, *df* = 1, *p* < .001) and suspended (*χ* ^2^ = 8.160, *df* = 1, *p* = .004) more frequently during the rainy season. Nevertheless, quadrupedal standing (*χ* ^2^ = 0.522, *df* = 1, *p* = .470) was not different between the rainy and dry seasons (Figure 2).

There were marked seasonal variations in the postures that langurs adopted during different activities. During feeding, langurs sat more frequently during the dry season (sitting: *χ* ^2^ = 9.830, *df* = 1, *p* = .002) but lay (*χ* ^2^ = 10.980, *df* = 1, *p* < .001) and suspended (*χ* ^2^ = 6.507, *df* = 1, *p* = .011) more frequently during the rainy season. However, the use of quadrupedal standing did not differ across the two seasons (*χ* ^2^ = 0.766, *df* = 1, *p* = .382). During resting, langurs sat more frequently (*χ* ^2^ = 11.690, *df* = 1, *p* < .001) in the dry season than in the rainy season, whereas they lay more frequently in the rainy season than in the dry season (*χ* ^2^ = 12.222, *df* = 1, *p* < .001). The frequencies of other resting postures did not differ by season (quadrupedal standing: *χ* ^2^ = 0.095, *df* = 1, *p* = .759; suspending: *χ* ^2^ = 0.189, *df* = 1, *p* = .664). During social grooming, langurs sat more frequently during the rainy season (*χ* ^2^ = 5.919, *df* = 1, *p* = .015), whereas they lay more frequently during the dry season (*χ* ^2^ = 5.877, *df* = 1, *p* = .015). The frequencies of quadrupedal standing (*χ* ^2^ = 1.032, *df* =1, *p* = .310) and suspending (*χ* ^2^ = 0.008, *df* = 1, *p* = .930) did not differ with season.

### 3.3 Substrate use

**FIGURE 3:**
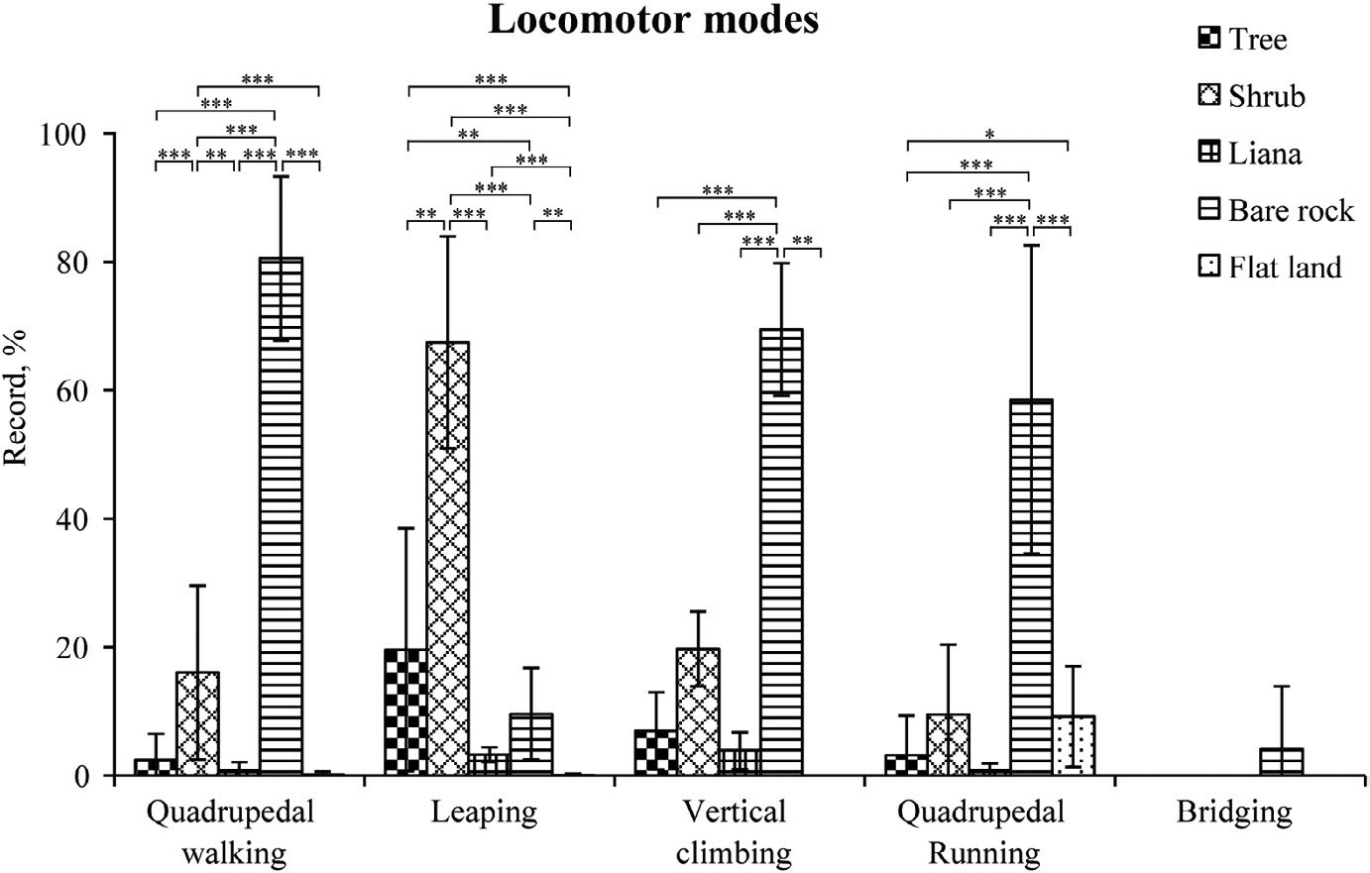
Frequencies of locomotor modes (based on substrates) used by the white‐headed langurs at Guangxi Chongzuo White‐Headed Langur National Nature Reserve from September 2016 to August 2017. Asterisks represent significant differences in proportions: ***p < .001; **p < .01; *p < .05

Langur locomotion differed across substrate types, except for bridging (Kruskal–Wallis test: quadrupedal walking: *χ* ^2^ = 158.89, *df* = 4, *p* < .001; leaping: *χ* ^2^ = 123.509, *df* = 4, *p* < .001; vertical climbing: *χ* ^2^ = 29.378 *df* = 4, *p* < .001; quadrupedal running: *χ* ^2^ = 73.074 *df* = 4, *p* < .001). Most locomotion occurred on shrubs and bare rocks. More specifically, quadrupedal walking (80.51% ± 12.78% of the records), vertical climbing (69.44% ± 10.33%), and quadrupedal running (58.55% ± 24.00%) occurred most frequently on bare rock. However, leaping most frequently occurred on shrubs (67.46% ± 16.52%; Figure 3).

**FIGURE 4:**
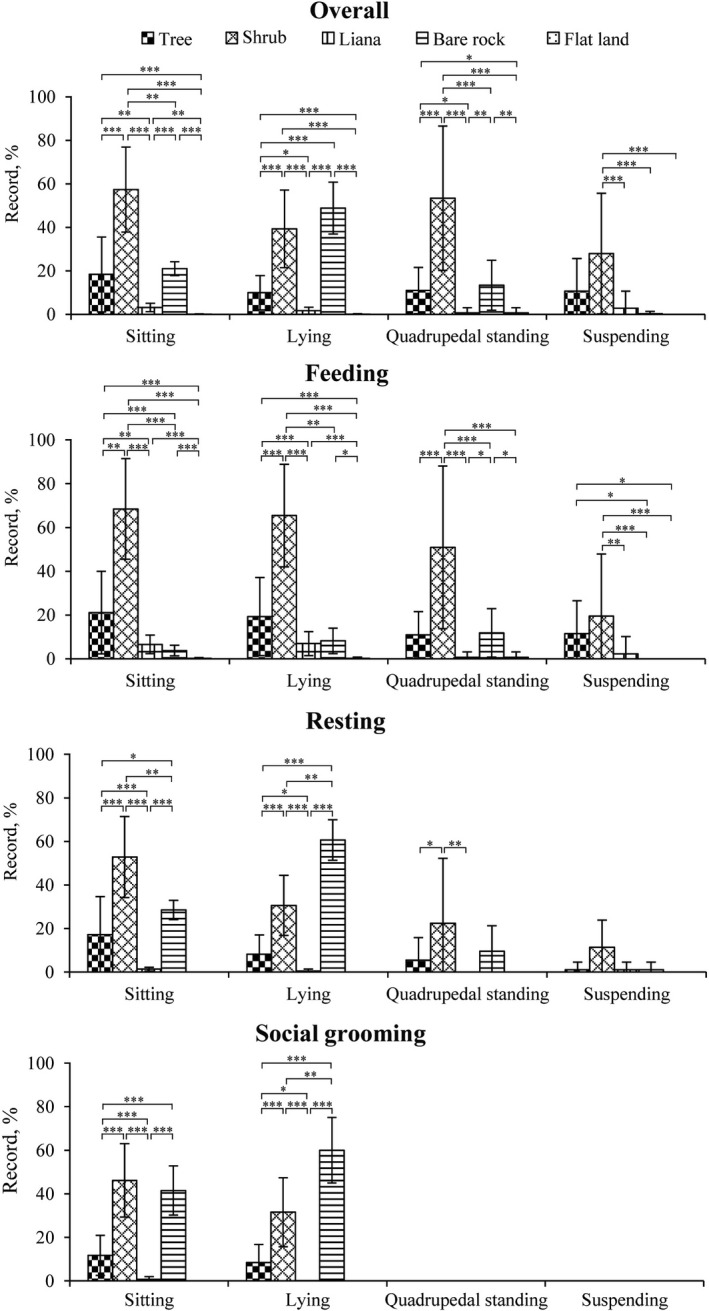
Frequencies of postures (based on substrates) during overall activity, resting, feeding, and social grooming used by the white‐headed langurs at Guangxi Chongzuo White‐Headed Langur National Nature Reserve from September 2016 to August 2017. Asterisks represent significant differences in proportions : ***p < .001; **p < .01; *p < .05

Langur posture differed by substrate type (Kruskal–Wallis test: sitting: *χ* ^2^ = 196.226, *df* = 4, *p* < .001; lying: *χ* ^2^ = 188.237, *df* = 4, *p* < .001; quadrupedal standing: *χ* ^2^ = 88.210, *df* = 4, *p* < .001; suspending: *χ* ^2^ = 39.573, *df* = 4, *p* < .001). The studied langurs predominantly utilized shrub or bare rock when stationary. During feeding, they most frequently used shrubs (sitting: 68.46% ± 22.95%; lying: 65.47% ± 23.44%; quadrupedal standing: 50.90% ± 37.16%). When resting, they sat more on shrub than on bare rock (52.83% ± 18.57% versus 28.55% ± 4.40%) but laid more on bare rock than on shrub (60.66% ± 9.32% versus 30.61% ± 13.78%). During social grooming, they laid more on bare rock than on shrub (60.00% ± 15.00% versus 31.56% ± 15.85%) but sat on shrub and bare rock in a similar frequency (46.16% ± 16.86% versus 41.49% ± 11.28%; Figure 4).

### 3.4 Effects of ecological factors on positional behavior

The model indicated that fruit availability and day length were the most important factors affecting the locomotor modes of white‐headed langurs (Table 3); the parameter estimates (*β*) of model‐averaged 95% confidence intervals for these variables did not contain zero. More specifically, quadrupedal walking increased with the decrease in fruit availability (*β* = −0.550, W*_i_* = 0.63), whereas leaping increased when fruit availability increased (*β* = 0.223, W*_i_* = 0.66). Moreover, quadrupedal running increased with day length (*β* = 39.774, W*_i_* = 0.71). Conversely, these ecological factors had no observable effect on vertical climbing or bridging (Table 3).

**TABLE 3:** Effect of ecological factors on positional behavior of the white‐headed langurs, based on the results of model averaging

Response variables	Variables	*β*	*SE*	*z*	*p*	95% CI		W*_ip_*
						Min	Max	
Quadrupedal walking	Young leave availability	−0.718	0.740	0.962	.336	−2.182	0.745	0.17
	Flower availability	−0.055	0.067	0.801	.423	−0.190	0.080	0
	**Fruit availability**	**−0.550**	**0.206**	**2.617**	.**009**	**−0.962**	**−0.138**	**0.63**
	Mature leave availability	0.046	1.912	0.024	.981	−3.730	3.823	0.33
	Rock maximum temperature	−1.727	1.267	1.335	.182	−4.264	0.809	0.52
	Forest average temperature	0.371	3.048	0.121	.904	−5.641	6.383	0.38
	Rainfall	−0.399	0.319	1.241	.215	−1.029	0.231	0.11
	Rock relative humidity	−0.274	0.590	0.457	.648	−1.451	0.902	0.17
	Forest relative humidity	−0.391	0.484	0.795	.426	−1.355	0.573	0.15
	Day length	3.809	4.536	0.826	.409	−5.227	12.846	0.62
Leaping	Young leave availability	−0.153	0.142	1.049	.294	−0.439	0.133	0.07
	Flower availability	−0.008	0.030	0.251	.802	−0.068	0.053	0
	**Fruit availability**	**0.223**	**0.090**	**2.423**	.**015**	**0.043**	**0.403**	**0.66**
	Mature leave availability	0.335	0.419	0.784	.433	−0.502	1.172	0.17
	Rock maximum temperature	−0.372	0.539	0.677	.498	−1.451	0.706	0.20
	Forest average temperature	−0.238	0.604	0.387	.699	−1.442	0.967	0.18
	Rainfall	−0.008	0.074	0.110	.912	−0.157	0.140	0.01
	Rock relative humidity	−0.085	0.220	0.376	.707	−0.525	0.356	0.07
	Forest relative humidity	−0.206	0.223	0.907	.365	−0.650	0.239	0.16
	Day length	−0.865	1.780	0.480	.631	−4.398	2.668	0.56
Vertical climbing	Young leave availability	0.308	0.308	1.514	.130	−0.091	0.706	0.24
	Flower availability	0.029	0.029	0.703	.482	−0.052	0.110	0
	Fruit availability	−0.050	−0.050	0.348	.728	−0.331	0.231	0.01
	Mature leave availability	−0.011	−0.011	0.017	.987	−1.275	1.254	0.16
	Rock maximum temperature	0.780	0.780	1.148	.251	−0.551	2.111	0.33
	Forest average temperature	0.715	0.715	0.947	.344	−0.765	2.195	0.38
	Rainfall	0.067	0.067	0.543	.587	−0.174	0.307	0.01
	Rock relative humidity	0.193	0.193	0.663	.507	−0.376	0.761	0.08
	Forest relative humidity	0.252	0.252	1.124	.261	−0.188	0.692	0.11
	Day length	−2.031	−2.031	0.963	.336	−6.168	2.105	0.53
Quadrupedal running	Young leave availability	−1.341	1.507	0.866	.386	−4.376	1.693	0.37
	Flower availability	−0.165	0.215	0.741	.459	−0.600	0.271	0
	Fruit availability	−0.541	0.793	0.662	.508	−2.140	1.059	0.24
	Mature leave availability	−1.379	4.085	0.330	.742	−9.578	6.820	0.52
	Rock maximum temperature	−2.813	4.067	0.671	.502	−11.024	5.399	0.58
	Forest average temperature	−9.811	5.423	1.763	.078	−20.718	1.095	0.71
	Rainfall	−0.021	0.779	0.026	.979	−1.587	1.545	0.14
	Rock relative humidity	−0.277	1.712	0.157	.875	−3.727	3.173	0.32
	Forest relative humidity	−0.984	1.238	0.772	.440	−3.483	1.515	0.35
	**Day length**	**39.774**	**12.228**	**3.170**	.**002**	**15.182**	**64.367**	**0.71**
Bridging	Young leave availability	−0.348	0.443	0.766	.444	−1.240	0.543	0.14
	Flower availability	−0.003	0.087	0.035	.972	−0.178	0.172	0
	Fruit availability	−0.108	0.282	0.374	.709	−0.674	0.458	0.03
	Mature leave availability	0.629	1.128	0.543	.587	−1.638	2.895	0.29
	Rock maximum temperature	1.255	1.538	0.795	.426	−1.838	4.349	0.41
	Forest average temperature	−1.766	1.535	1.123	.262	−4.849	1.317	0.49
	Rainfall	0.011	0.220	0.048	.962	−0.431	0.453	0.01
	Rock relative humidity	0.282	0.648	0.424	.671	−1.021	1.585	0.16
	Forest relative humidity	0.041	0.441	0.091	.927	−0.847	0.930	0.08
	Day length	−1.673	3.982	0.411	.681	−9.660	6.314	0.54
Sitting	Young leave availability	0.295	0.183	1.577	.115	−0.072	0.661	0.26
	**Flower availability**	**0.066**	**0.029**	**2.248**	.**025**	**0.008**	**0.124**	**0.13**
	Fruit availability	0.063	0.101	0.610	.542	−0.140	0.266	0.03
	Mature leave availability	0.182	0.538	0.334	.739	−0.887	1.251	0.17
	Rock maximum temperature	0.425	0.563	0.737	.461	−0.705	1.554	0.26
	**Forest average temperature**	**2.159**	**0.581**	**3.642**	.**000**	**0.997**	**3.321**	**0.88**
	Rainfall	−0.045	0.095	0.469	.639	−0.235	0.144	0.03
	Rock relative humidity	−0.327	0.220	1.452	.146	−0.769	0.114	0.26
	Forest relative humidity	0.189	0.166	1.110	.267	−0.144	0.522	0.11
	**Day length**	**−8.710**	**1.423**	**5.982**	**<2e−16**	**−11.563**	**−5.856**	**0.89**
Lying	Young leave availability	−0.340	0.185	1.798	.072	−0.710	0.031	0.33
	**Flower availability**	**−0.068**	**0.029**	**2.323**	.**020**	**−0.126**	**−0.011**	**0.15**
	Fruit availability	−0.023	0.107	0.211	.833	−0.237	0.191	0.01
	Mature leave availability	−0.290	0.549	0.522	.602	−1.381	0.800	0.19
	Rock maximum temperature	−0.445	0.576	0.755	.450	−1.602	0.711	0.27
	**Forest average temperature**	**−1.976**	**0.612**	**3.169**	.**002**	**−3.198**	**−0.754**	**0.82**
	Rainfall	0.080	0.085	0.918	.358	−0.090	0.250	0.06
	Rock relative humidity	0.320	0.223	1.402	.161	−0.127	0.767	0.22
	Forest relative humidity	−0.161	0.170	0.924	.355	−0.502	0.180	0.07
	**Day length**	**8.476**	**1.400**	**5.916**	**<2e−16**	**5.668**	**11.284**	**0.84**
Quadrupedal standing	Young leave availability	−1.052	1.855	0.560	.576	−4.737	2.632	0.59
	Flower availability	−0.007	0.159	0.045	.964	−0.329	0.314	0
	Fruit availability	−1.315	0.713	1.805	.071	−2.743	0.113	0.55
	Mature leave availability	3.703	5.157	0.710	.478	−6.525	13.931	0.69
	**Rock maximum temperature**	**−7.216**	**3.037**	**2.308**	.**021**	**−13.344**	**−1.088**	**0.86**
	Forest average temperature	−6.880	7.979	0.853	.394	−22.696	8.936	0.74
	Rainfall	−1.250	0.637	1.925	.054	−2.523	0.023	0.64
	Rock relative humidity	0.186	1.618	0.113	.910	−3.047	3.419	0.38
	Forest relative humidity	0.635	1.103	0.561	.575	−1.583	2.852	0.33
	**Day length**	**34.830**	**11.716**	**2.911**	.**004**	**11.381**	**58.280**	**0.86**
Suspending	Young leave availability	−0.144	1.331	0.106	.915	−2.793	2.505	0.30
	Flower availability	−0.060	0.132	0.439	.660	−0.327	0.207	0
	**Fruit availability**	**−1.299**	**0.460**	**2.745**	.**006**	**−2.226**	**−0.371**	**0.83**
	Mature leave availability	0.861	3.194	0.265	.791	−5.497	7.219	0.56
	Rock maximum temperature	−0.567	2.549	0.216	.829	−5.708	4.574	0.51
	**Forest average temperature**	**−11.696**	**4.083**	**2.813**	.**005**	**−19.845**	**−3.546**	**0.86**
	Rainfall	−0.545	0.547	0.979	.328	−1.635	0.546	0.23
	Rock relative humidity	−0.142	1.134	0.123	.902	−2.419	2.134	0.30
	Forest relative humidity	−0.024	0.907	0.026	.979	−1.847	1.798	0.25
	**Day length**	**45.777**	**9.201**	**4.858**	.**000**	**27.309**	**64.244**	**0.86**

Note

Model‐averaged 95% confidence intervals excluded zero listed in bold.

Abbreviations: 95% CI, the 95% confidence intervals for *β*; Wip, relative variable importance; *β*, model‐averaged regression coefficients.

Postures were significantly influenced by fruit availability, average temperature, and day length. The parameter estimates of the model‐averaged 95% confidence intervals of the variable also excluded zero. More specifically, sitting frequency was positively correlated with average temperature (*β* = 2.159, W*_i_* = 0.88) but negatively correlated with day length (*β* = −8.710, W*_i_* = 0.89). Contrarily, lying decreased with the increase in average temperature (*β* = −1.976, W*_i_* = 0.82) but increased as day length increased (*β* = 8.476, W*_i_* = 0.84). Quadrupedal standing was negatively correlated with maximum temperature (*β* = −7.216, W*_i_* = 0.86) but positively correlated with day length (*β* = 34.830, W*_i_* = 0.86). Suspending decreased with the increase in fruit availability (*β* = −1.299, W*_i_* = 0.83) and average temperature (*β* = −11.696, W*_i_* = 0.86), but it was positively correlated with day length (*β* = 45.777, W*_i_* = 0.86; Table 3).

## 4 DISCUSSION

### 4.1 Effects of limb length, body size, and tail length

Leaping and vertical climbing were the dominant locomotor modes for white‐headed langurs, which is consistent with prediction 1. Generally, the leaping ability of animals is related to the species’ morphological and anatomical characteristics, such as limb length, body size, and tail length (Chatani, 2003; Fleagle, 2013; Table 4). In this study of white‐headed langurs, leaping was the most dominant locomotor mode, which may be partly attributed to their lower IMI and small body size (Pan et al., 1989). When leaping, most of the propulsive forces are generated from the hind limbs, and larger individuals must generate greater forces than smaller individuals (Fleagle, 2013). Therefore, smaller species were more likely to adopt leaping to cross discontinuities (Fleagle & Meldrum, 1988). The frequency of vertical climbing can be partly influenced by body mass, because vertical climbing is energetically expensive for larger individuals, as reported in chimpanzees (*Pan troglodytes*; Hunt, 1994). However, langurs have a small body size, which may partially contribute to the high frequency of vertical climbing. Limb length is correlated with primates’ leaping ability (Fleagle, 2013). Primates with lower IMIs are skilled at leaping, such as François’ langurs (Chen et al., 2020) and Indo‐Chinese gray langurs (*T. crepusculus*; Ma & Fan, 2020). Contrarily, those with a higher IMI are skilled in suspensory locomotion, such as Cao Vit gibbons (*Nomascus nasutus*; Fan et al., 2013). Other species with an intermediate IMI tend to travel in a quadrupedal manner (Chatani, 2003; Fleagle & Meldrum, 1988; Table 4), such as Assamese macaques (*M*. *assamensis*), rhesus macaques (*M. mulatta*; Huang, Huang, Wei, et al., 2015), and Japanese macaques (*M. fuscata*; Chatani, 2003). Moreover, white‐headed langurs have a long tail (Pan et al., 1989) that may help in balancing and enhance their leaping ability (Cant, 1988; Chatani, 2003).

**TABLE 4:** Locomotor modes of several colobines

Species	Study site ^a^	Sampling method^b^	IMI	Body mass		Quadrupedalism	Leaping	Climbing	Other	Ref.
				Male	Female					
*Trachypithecus francoisi*	1	S	83.0	8.0	7.8	36.5	38.4	25.1	0.0	Chen et al. (2020)
	1	S	–	7.1	6.7	34.1	46.3	13.4	6.2	Xiong et al. (2009)
	1	S	–	–	–	31.1	43.3	25.5	0.0	Zhou et al. (2013)
*T. leucocephalus*	1	S	–	8.8	7.8	30.6	47.3	19.7	2.4	Xiong et al. (2009)
	1	S	–	–	–	11.9	47.9	40.1	<0.1	This study
	1	F	75.4–76.4	8.8	7.8	66.8	12.6	20.6	0.0	Huang and Li (2005)
*T. delacouri*	2	F	77.0	8.6	7.8	63.8	7.5	26.3	2.5	Workman and Schmitt (2012)
*T. obscurus*	3	F	83.0	7.9	6.4	50.6	40.2	9.2	0.0	Fleagle (1980)
*T. crepusculu*	4	S	–	–	–	26.1	58.0	15.2	0.6	Ma and Fan (2020)
*Colobus badius*	5	F	87.0	8.3	8.2	37.0	25.0	32.0	6.0	Gebo and Chapman (1995b)
*C. guereza*	5	F	79.0	10.1	8.0	41.0	38.0	15.0	6.0	Gebo and Chapman (1995b)
*Rhinopithecus roxellana*	6	S	–	–	–	47.4	26.7	19.8	6.1	Zhu et al. (2015)

^a^ Study site: 1—Southwest China, limestone forest; 2—Northern Vietnam, limestone forest; 3—Peninsular Malaysia, nonlimestone forest; 4—Wuliang Mountain, nonlimestone forest; 5—Western Uganda, nonlimestone forest; 6—Qinling Mountains, nonlimestone forest.

^b^ Sampling method: S—scan sampling; F—focal animal sampling.

White‐headed langurs predominantly sat when stationary. It is common for those colobines to spend the majority of their time sitting (Table 5). This may be related to their ischial callosities, which can assist in long periods of sitting (Napier, 1967). There are many sharp, razor‐like points on the surface of karst limestone mountains (Workman & Schmitt, 2012), such that sitting on ischial callosities is extremely crucial for these limestone forest primates, as reported in François’ langurs (Chen et al., 2020) and Delacour's langurs (Workman & Schmitt, 2012).

**TABLE 5:** Postures of several colobines

Species	Study site^a^	Sampling method^b^	Sitting	Lying	Standing	Suspending	Other	Ref.
*Trachypithecus francoisi*	1	S	92.1	3.5	4.3	0.2	0	Chen et al. (2020)
*T. leucocephalus*	1	S	83.1	16.2	0.2	0.6	0	This study
*T. delacouri*	2	F	95	2	3	0	0	Workman and Schmitt (2012)
*T. crepusculu*	3	S	94.8	4.6	0.5	0.1	0	Ma and Fan (2020)
*Colobus badius*	4	F	90	1	8	< 2	< 1	Gebo and Chapman (1995b)
*C. guereza*	4	F	87	8	4	< 2	< 1	Gebo and Chapman (1995b)
*Rhinopithecus roxellana*	5	S	87.3	0.1	9.5	2.6	0.4	Zhu et al. (2015)

^a^ Study site: 1—Southwest China, limestone forest; 2—Northern Vietnam, limestone forest; 3—Wuliang Mountain, nonlimestone forest; 4—Western Uganda, nonlimestone forest; 5—Qinling Mountains, nonlimestone forest.

^b^ Sampling method: S—scan sampling; F—focal animal sampling.

### 4.2 Effect of habitat structure

In this study, locomotor patterns were related to habitat structure (Table 4). Leaping was the most frequently used locomotor mode for white‐headed langurs, and it predominantly occurred on shrubs (Figure 3). In the limestone forest, trees are vertically distributed but are not contiguous, resulting in many gaps that the primates inhabiting the limestone forest must cross (Fan et al., 2011). Leaping is commonly linked to crossing open spaces within canopies (Gebo & Chapman, 1995b); therefore, leaping would be an effective method for white‐headed langurs to cross these gaps. Similar patterns have been observed in the locomotor modes of François’ langurs (Chen et al., 2020; Zhou et al., 2013). Even in nonlimestone forests, which are characterized by many gaps within tree canopies and unstable liana substrates, primates have also exhibited similar patterns (Ma & Fan, 2020), as is the case in Indo‐Chinese gray langurs (Ma & Fan, 2020).

The locomotor modes of limestone forest‐dwelling langurs exhibit a dramatic variation. For instance, Delacour's langurs (*T. delacouri*), an endangered primate in Vietnam, primarily adopt quadrupedal locomotion while traveling and rarely use leaping (Workman & Schmitt, 2012), which differs from the locomotor pattern of white‐headed langurs. In this study, the white‐headed langurs leaped more frequently than Delacour's langurs (47.9% versus 7.5%; Workman & Schmitt, 2012); such differences may be due to the obvious differences in the forest structure between Vietnam and Southwestern Guangxi, China. In the habitat of Delacour's langurs, vegetation is sparse and stunted, and few trees are strong enough to support the langurs’ weights (Workman & Schmitt, 2012). The habitat of the white‐headed langur is severely fragmented, vegetation grows well, and trees are dominant on the middle and bottom of the hills. This may lead to differences in the locomotor modes between the two species. Moreover, our study group moved as quadrupedalism more frequently than previous studies (Huang & Li, 2005; Xiong et al., 2009). This pattern should be correlated to the structure of vegetation structure. Although we have no comparative data on the vegetation structure of all study sites, the habitats of white‐headed langurs live in the habitats with more abundant and taller trees than those of previous study conducted in the 1990s (unpublished data; Huang & Li, 2005; Xiong et al., 2009), likely allowing langurs less frequently quadrupedal traveling on the bare rocks.

In this study, white‐headed langurs adopted vertical climbing as the second most important locomotor mode, which predominantly occurred on bare rocks (Figure 3). This may be because there is a large area of vertical cliffs in limestone forests, and langurs choose ledges and caves in the middle and top of vertical cliffs as their sleeping sites (Chen, 2011; Huang, 2002). Therefore, climbing may be a relatively effective locomotor mode for karst‐dwelling primates to travel across cliffs (Huang, Huang, Wei, et al., 2015). Other sympatric primates, such as François’ langurs (Chen et al., 2020; Zhou et al., 2013) and Assamese macaques (Huang, Huang, Wei, et al., 2015), also choose ledges and caves as their sleeping sites and spend much of their time climbing along cliffs. Compared with limestone forest primates, other primates inhabiting forested habitats spend less time climbing (Table 4), such as Indo‐Chinese gray langurs (Ma & Fan, 2020).

### 4.3 Effect of food availability, temperature, and day length

Our results indicate that white‐headed langurs used quadrupedal walking more frequently during the dry season than during the rainy season. Moreover, quadrupedal walking was negatively correlated with the monthly fruit availability (*β* = −0.550, W*_ip_* = 0.63). This is consistent with prediction 2. Previous studies found that white‐headed langurs select food depending on plant availability (Lu et al., 2016). Moreover, these langurs significantly increase foraging time to obtain adequate foods (Huang, 2002; Zhou et al., 2010) when the availability of preferred food decreases during the dry season (Huang, 2002). Quadrupedal walking is an energetically inexpensive locomotor mode (Nakatsukasa et al., 2006; Youlatos, 1998). Thus, they may walk more frequently, which helps minimize energy consumption and maximize foraging benefits. In fact, the white‐headed langurs have an energy‐saving strategy in response to a reduction in the preferred food items, including having shorter daily traveling distance (Zhou et al., 2011) and spending more time moving and feeding during dry and/or cold months (Zhou et al., 2010). A similar pattern is observed in red colobus monkeys (*Colobus badius*; Gebo & Chapman, 1995b), red howling monkeys (Youlatos, 1998), and François’ langurs (Chen et al., 2020).

Moreover, leaping was positively linked to fruit availability (*β* = 0.223, W*_ip_* = 0.66). White‐headed langurs tend to depend on young leaves while still preferring fruits (Huang et al., 2017; Li & Rogers, 2006). Compared with leaves, fruits contain more simple sugars, which are generally easier to digest, can be rapidly converted into energy, and are considered to be high‐quality food items (Richard, 1985). This may explain why langurs exhibit a distinctive preference for fruits when they are available, likely providing adequate energy that was spent in traveling. In addition, fruits are predominantly distributed in the discontiguous canopies (Youlatos, 1998). Thus, when fruit is highly available, these langurs may leap more frequently between canopies for fruits to harvest more energy. Indeed, when high‐quality foods are available, animals can adopt a “high‐cost, high‐benefit” energy balance strategy to achieve a maximum net energy gain (Li et al., 2020a).

The postures of primates are strongly influenced by temperature (Bicca‐Marques & Calegaro‐Marques, 1998; Chen et al., 2020; Donati et al., 2011; Kelley et al., 2016). In this study, white‐headed langurs sat more but laid less during the dry season than during the rainy season, which is consistent with prediction 3. Temperatures are a vitally influential factor to sitting and lying (Bicca‐Marques & Calegaro‐Marques, 1998; Chen et al., 2020). At our study site, the ambient temperature during the dry season was lower than that during the rainy season (Huang et al., 2017; Zhou et al., 2010). Besides, the limestone forest is characterized by a carbonatite matrix, resulting in a lower bare rock surface temperature during the dry season and a higher temperature than that under the tree crown during the rainy season (Huang, 2002). Therefore, white‐headed langurs like to sit together in huddles or sunbathe to conserve energy in the cold season, as is reported in red‐collared lemurs (*Eulemur collaris*; Donati et al., 2011) and ring‐tailed lemurs (*Lemur catta*; Kelley et al., 2016). However, it was surprising that they decreased the frequency of sitting during social grooming in the dry season. This is likely because lying down in the sunshine can facilitate adequate grooming and increase the surface area to gain more heat, whereas during the rainy season, primates are able to stretch themselves out and spend more time shading for the purpose of heat avoidance (Dasilva, 1993; McFarland et al., 2020). Therefore, langurs like to stretch themselves out to dissipate heat in the shade or in caves during the rainy season (Chen et al., 2020; Huang, 2002).

Postures were also influenced by day length. Seasonal variations in day length strongly affect primate behavior (van Doorn et al., 2010; Hill et al., 2003; Ren et al., 2009), particularly for social activities (e.g., grooming and huddling). Generally, social touch plays a crucial role in maintaining social relationships (Dunbar et al., 2009). For instance, as day length decreases, sympatric Assamese macaques increase their social grooming and reduce their movement (Li, Ma, Zhou, & Huang, 2020). This pattern may be linked to their need to establish stabilized social groups. During shorter days (dry season), in this study, white‐headed langurs increased sitting together (cowering and huddling) for social interactions (Table 3). Moreover, sitting together in huddles contributes to the reduction in heat dissipation in the dry months. In other words, although nutrition and energy satisfaction are essential to survival, there is also a need for sufficient social interaction to strengthen group stability (Dunbar et al., 2009; Li, Ma, Zhou, & Huang, 2020).

In summary, white‐headed langurs engaged in leaping and vertical climbing as their predominant locomotor modes in limestone forests. Locomotor modes and postures showed significant seasonal variations. Langurs adopted quadrupedal walking more frequently during the dry season than during the rainy season. Overall, they sat more frequently in the dry season and lay more frequently in the rainy season, during both resting and feeding; however, during social grooming, they demonstrated the opposite pattern. The positional behavior of white‐headed langurs was affected by their morphological and anatomical characteristics, especially limb length. Seasonal variation in positional behavior may be linked to seasonal variation in food availability and their needs in terms of behavioral thermoregulation and group stability. When food availability was lower during the dry season, langurs adopted quadrupedal walking more frequently for the purpose of reducing the energy expenditure associated with foraging. When the day length was shorter during the dry season (cold season), they huddled together or sunbathed to conserve energy and stabilize the group. These results indicate that white‐headed langurs adapt to limestone forests with positional behavior flexibility in response to seasonality and habitat structure. Our research provides evidence of the effects of food availability, ambient temperature, and day length on the positional behavior of white‐headed langurs, highlighting the need to increase our understanding of their behavioral ecology and the influences of ecological factors on behavioral adaptation.

## CONFLICT OF INTEREST

None declared.

## AUTHOR CONTRIBUTIONS

**Jingjin Zheng:** Formal analysis (equal); Writing‐original draft (lead); Writing‐review & editing (equal). **Kechu Zhang:** Investigation (lead). **Jipeng Liang:** Investigation (supporting). **Youbang Li:** Formal analysis (equal); Funding acquisition (supporting); Writing‐review & editing (equal). **Zhonghao Huang:** Conceptualization (lead); Formal analysis (equal); Funding acquisition (lead); Methodology (lead); Project administration (equal); Supervision (lead); Writing‐review & editing (equal).

## Data Availability

All data are available in the figshare repository at https://figshare.com, with the DOI https://doi.org/10.6084/m9.figshare.15167715.v1
